# Laparoscopic and open surgery in rectal cancer patients in Germany: short and long-term results of a large 10-year population-based cohort

**DOI:** 10.1007/s00464-019-06861-4

**Published:** 2019-05-30

**Authors:** Valentin Schnitzbauer, Michael Gerken, Stefan Benz, Vinzenz Völkel, Teresa Draeger, Alois Fürst, Monika Klinkhammer-Schalke

**Affiliations:** 1 Faculty of Medicine — University Hospital Regensburg, University of Regensburg, Franz-Josef-Strauß-Allee 11, 93053 Regensburg, Germany; 2 Tumor Center Regensburg, Institute for Quality Assurance and Health Services Research, University of Regensburg, Am BioPark 9, 93053 Regensburg, Germany; 3 Klinik für Allgemeine-,Viszeral- und Kinderchirurgie, Kliniken Böblingen, Bunsenstr. 120, 71032 Böblingen, Germany; 4 Department of Surgery, Caritas Clinic St. Josef, Landshuter Strasse 65, 93053 Regensburg, Germany; 5 Arbeitsgemeinschaft Deutscher Tumorzentren e.V., Kuno-Fischer-Strasse 8, 14057 Berlin, Germany

**Keywords:** Minimal invasive surgery, Rectal cancer, Long-term survival, Short-term survival, Retrospective analysis, Health services research

## Abstract

**Background:**

Rectal cancer is frequent in Germany and worldwide. Several studies have assessed laparoscopic surgery as a treatment option and most have shown favorable results. However, long-term oncologic safety remains a controversial issue.

**Methods:**

The current dataset derives from 30 clinical cancer registries in Germany and includes 16,378 patients diagnosed with rectal cancer between 2007 and 2016. Outcomes were 90-day mortality, overall survival (OS), local recurrence-free survival (RFS) and relative survival of patients treated with either open or laparoscopic surgery. Multivariable logistic regression was used to evaluate factors that affected the probability of a patient undergoing laparoscopic surgery as well as to evaluate short-term mortality. OS and RFS were analyzed by Kaplan–Meier plots and multivariable Cox regression conducted separately for UICC stages I–III, tumor location, and sex as well as by propensity score matching followed by univariable and multivariable survival analysis.

**Results:**

Of 16,378 patients, 4540 (27.7%) underwent laparoscopic surgery, a trend which increased during the observation period. Patients undergoing laparoscopy attained better results for 90-day mortality (odds ratio, OR 0.658, 95% confidence interval, CI 0.526–0.822). The 5-year OS rate in the laparoscopic group was 82.6%, vs. 76.6% in the open surgery group, with a hazard ratio (HR) of 0.819 in multivariable Cox regression (95% CI 0.747–0.899, *p* < 0.001). The laparoscopic group showed a better 5-year RFS, with 81.8 vs. 74.3% and HR 0.770 (95% CI 0.705–0.842, *p* < 0.001). The 5-year relative survival rates were also in favor of laparoscopy, with 93.1 vs. 88.4% (*p* = 0.012).

**Conclusion:**

Laparoscopic surgery for rectal cancer can be performed safely and, according to this study, is associated with an oncological outcome superior to that of the open procedure. Therefore, in the absence of individual contraindications, it should be considered as a standard approach.

Colorectal cancer (CRC) is one of the most frequent malignant diseases worldwide. In Germany, it is the third most common cancer in men and the second most common in women [1]. CRC is also considered to be one of the most common cancers in the USA [2]. Minimally invasive surgical techniques have been described in the literature for the past three decades, with a history dating back to the introduction of laparoscopic cholecystectomy in 1985, marking a significant shift from open surgery [3]. Since the first successful application of laparoscopy in CRC patients in 1991, the technique as well as the instruments have improved. Surgeons have progressed along a steep learning curve, leading to a decrease in surgical complications [4, 5]. Laparoscopic treatment has been compared to open surgery separately for colon and rectal cancer, as well as for these entities combined in CRC. For colon cancer, randomized trials, population-based studies, and meta-analyses have generally shown positive results. Beneficial effects of laparoscopy in terms of 30-day mortality have been found in population-based studies in England, France, and the US [6–8]. German registry-based studies with a large number of patients found laparoscopy to be an independent predictor of better long-term survival [9], especially in patients with low-risk colon cancer [10]. However, different meta-analyses could not confirm significant differences for OS or RFS upon comparing the two surgical approaches [11–14]. Fewer studies have been conducted regarding rectal cancer, but there are findings that indicate equivalence and partly superiority of laparoscopy over open surgery for rectal cancer in short-term follow-up. According to the high-quality COLOR II study, long-term oncologic outcome for laparoscopy is promising [15]. Moreover, in a German population-based study, the beneficial effect of minimally invasive surgery on 5-year local recurrence-free survival was found to be highly significant [16]. The aim of the current study is to add evidence to the field by conducting a nationwide analysis comparing laparoscopic to open surgery for rectal cancer, with primary focus on long-term OS and RFS.

## Materials and methods

The pooled database used in this study consists of 30 separate data packages provided by the Association of Clinical Cancer Registries in Germany (ADT). The purpose of such regional registries is to collect data on cancer patients to reveal diagnostic or therapeutic shortcomings with the intention of improvement. Registries in the south and east of Germany are overrepresented in the dataset, because the variables considered necessary for this study have been collected in these regions for a longer period of time. For the present study, all registered rectal cancer cases from 2007 to 2016 with information on the surgical approach were considered. Data contain explicit details about sex, age, tumor location, histologic type, Union for International Cancer Control (UICC) stage, grading, surgery, and perioperative therapy, as well as on survival and recurrences. The classification of tumor location was carried out according to UICC specifications by measurement from the anocutaneous line (upper rectum: > 12–16 cm, middle rectum: > 6–12 cm, lower rectum: < 6 cm). All patient data were anonymized. The study design was reviewed and approved by the Ethical Review Board of the University of Regensburg, Germany (approval no. 15-170-0000).

### Patient collective

**Fig. 1:**
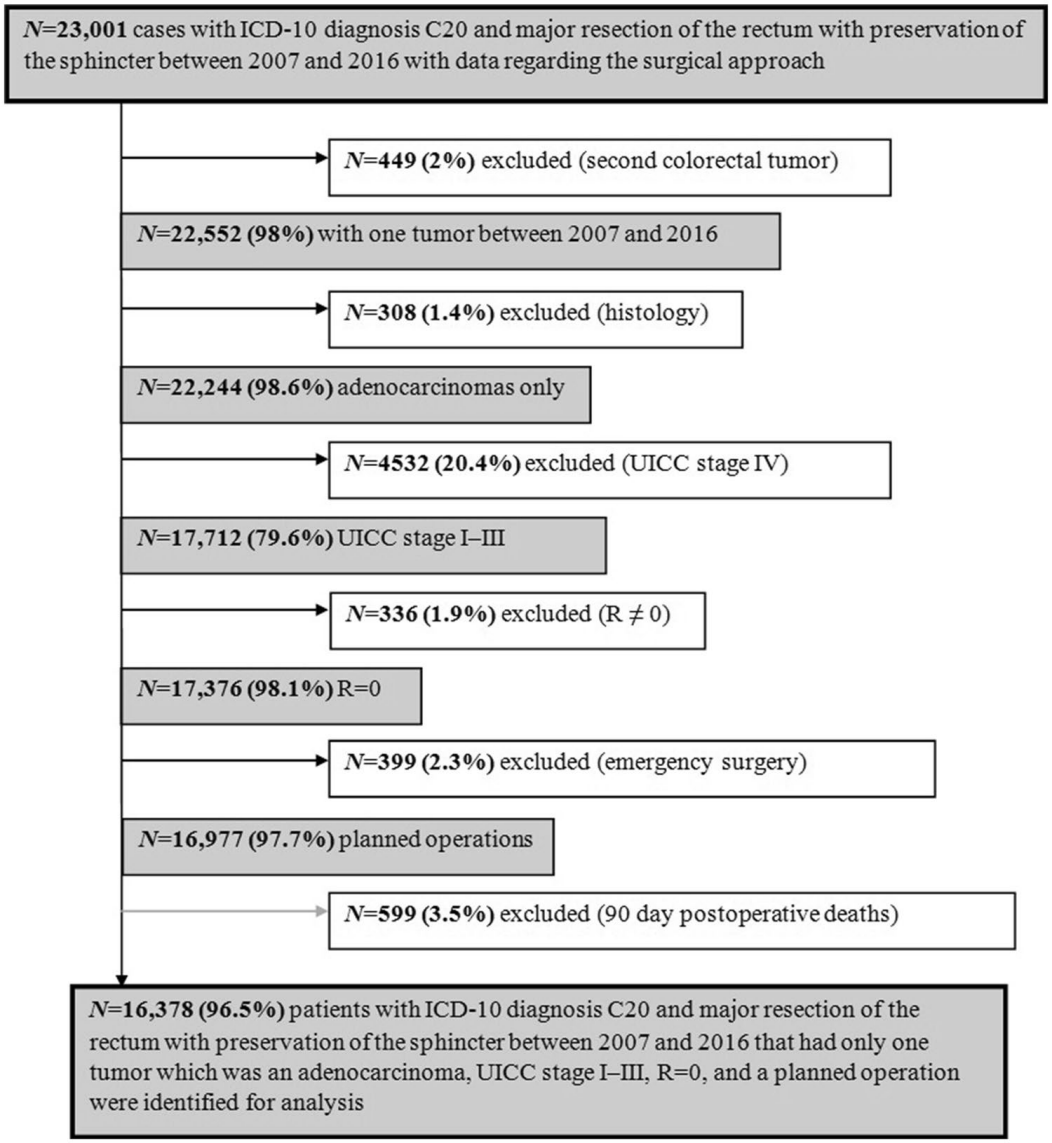
Flowchart of study patient selection

23,001 patients with rectal cancer (ICD-10 C20) who had undergone either laparoscopic or open surgery with sphincter preservation between 2007 and 2016 were identified. Patients with a second previous or simultaneous colorectal tumor, or with histological types other than adenocarcinoma were excluded (Fig. 1). Furthermore, only patients with UICC stages I–III who had undergone R0 resection were included. Emergency surgery cases were ruled out and patients who died within 90 days after surgery were excluded from the analysis of long-term outcome to discriminate 90-day mortality from long-term survival. After applying the above mentioned criteria, 16,378 patients were available for long-term analyses.

### Statistical analysis

Metric variables were analyzed for differences in their mean values using student’s *t* test. Independence of categorical variables was analyzed using Pearson’s Chi squared test. Analyses were carried out on an intention-to-treat basis, which means that cases remained in the laparoscopic group even if the surgeon decided to switch to open surgery. This reduces the unfavorable effects of the more severe or more demanding cases that would otherwise go on the account of the open surgery approach. Imbalanced variables were considered to potentially induce bias in logistic regression and survival analyses, and were adjusted for in multivariable analyses. The likelihood of undergoing laparoscopic surgery was estimated via multivariable binary logistic regression and was reported as an odds ratio (OR). Multivariable logistic regression was also used to analyze short-term mortality for the whole study population, including the patients who died within 90 days after surgery, thus creating a slightly larger collective of 16,977 patients.

Univariable survival analyses for OS and RFS were performed using Kaplan–Meier and Cox regression methods in combination with the logrank test. Follow-up was estimated by means of reverse Kaplan–Meier-method. Multivariable survival analyses comparing the minimally invasive surgical approach with open surgery were done using the Cox proportional hazards model, adjusting for the same variables as in multivariable logistic regression.

Multivariable Cox regression was also used to analyze OS and RFS for UICC stages I–III individually. In addition, separate analyses for OS and RFS were performed for tumor location in the upper, middle, and lower rectum, and for male and female sex.

To balance patients’ characteristics between the two groups and to diminish bias in survival analyses, we additionally performed propensity score matching (1:2 nearest neighbor matching with caliper 0.2, balancing for sex, age, tumor location, stage, grading, and perioperative therapy), which rendered 4534 patients with laparoscopic and 8817 patients with open surgery (total 13,351 patients).

The results were reported with hazard ratios (HRs) and 95% confidence intervals (CIs). A *p* value < 0.05 was considered significant for all tests. Computing a relative survival model puts the survival of patients in the present study into the context of survival among the general population, which is estimated via mortality tables in accordance with the age and sex distribution. The underlying data for general mortality in Germany come from the Human Mortality Database of the Max Planck Institutes [17]. Analyses were performed using SPSS (version 25, IBM SPSS Statistics, Armonk, NY, USA) and R (version 3.3.2; R Foundation for Statistical Computing, Vienna; http://www.r-project.org/) with the R package “relsurv” (Maja Pohar-Perme [18]).

## Results

### Patient characteristics and determinants of the laparoscopic approach

**Fig. 2:**
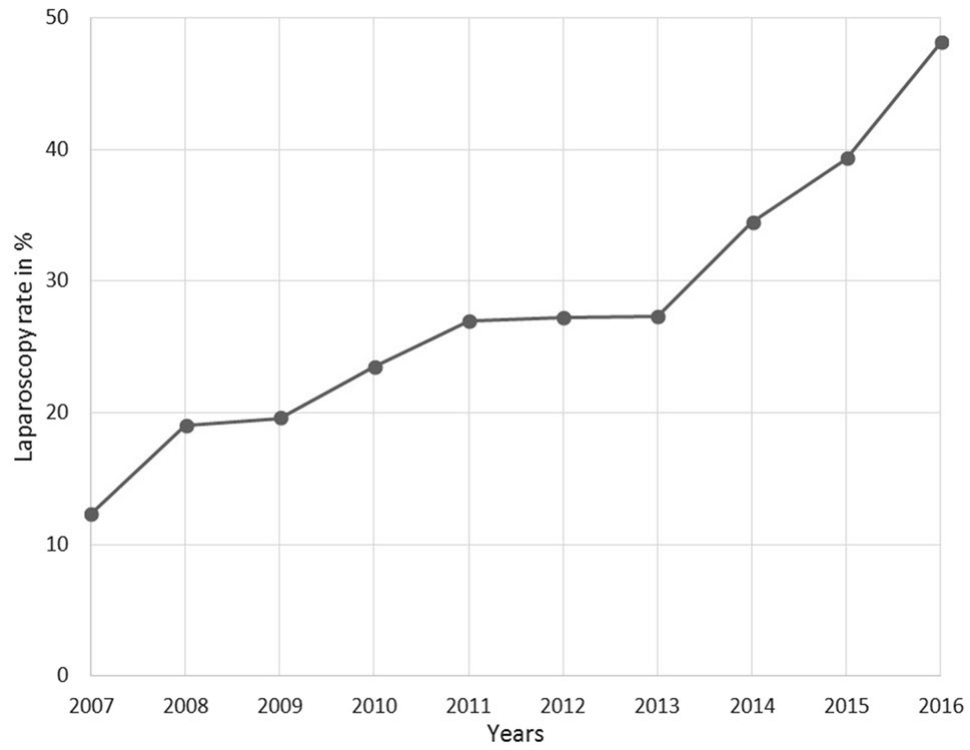
Use of the laparoscopic approach for treatment of rectal cancer

The proportion of patients undergoing laparoscopic surgery for rectal cancer was 27.7% (4540 patients), whereas 72.3% (11,838 patients) underwent open surgery. Within our dataset, the use of laparoscopy increased steadily from 12.3 to 48.1% between 2007 and 2016 (Fig. 2). There were 2731 men (60.2%) and 1809 women (39.8%) in the laparoscopic group, compared to 7627 men (64.4%) and 4211 women (35.6%) in the open surgery group (*p *< 0.001, Table 1). Patients’ mean age was 67.37 years, with significant differences between the treatment groups. Those who underwent laparoscopic surgery were 2.06 years younger than those who received open surgery (*p *< 0.001). The open surgery approach acts as a reference, with OR 1.000 for all following analyses. Multivariable logistic regression revealed that younger age (OR 0.982/year, 95% CI 0.978–0.985, *p *< 0.001), female sex (OR 1.245, 95% CI 1.157–1.340, *p *< 0.001), higher rectum location (OR 1.202, 95% CI 1.073–1.346, *p *< 0.002 for middle rectum and OR 1.476, 95% CI 1.306–1.668, *p *< 0.001 for upper rectum), low UICC stage (OR 0.742, 95% CI 0.677–0.813, *p *< 0.001 for UICC II and OR 0.845, 95% CI 0.776–0.920, *p *< 0.001 for UICC III), and low grading (OR 0.826, 95% CI 0.739–0.924, *p *< 0.001 for G3/4) were independent factors that led to a higher chance of receiving laparoscopy (Table 2).

**Table 1:** Patient characteristics

	Surgical approach						
	Laparoscopic		Open		Total		χ^2^
	*N*	%	*N*	%	*N*	%	*p* value
Sex							
Male	2731	60.2	7627	64.4	10358	63.2	< 0.001
Female	1809	39.8	4211	35.6	6020	36.8	
Age at diagnosis (years)							
0–49	392	8.6	680	5.7	1072	6.5	< 0.001
50–59	1015	22.4	2164	18.3	3179	19.4	
60–69	1319	29.1	3424	28.9	4743	29.0	
70–79	1376	30.3	4067	34.4	5443	33.2	
80+	438	9.6	1503	12.7	1941	11.9	
Location rectum							
Lower rectum	601	13.2	2076	17.5	2677	16.3	< 0.001
Middle rectum	1347	29.7	3863	32.6	5210	31.8	
Upper rectum	1228	27.0	2969	25.1	4197	25.6	
Unspecified	1364	30.0	2930	24.8	4294	26.2	
Stage UICC							
I	1692	37.3	3868	32.7	5560	33.9	< 0.001
II	1159	25.5	3589	30.3	4748	29.0	
III	1689	37.2	4381	37.0	6070	37.1	
Grading							
G1/2	3661	80.6	9528	80.5	13189	80.5	< 0.001
G3/4	495	10.9	1617	13.7	2112	12.9	
Unspecified	384	8.5	693	5.9	1077	6.6	
Radio-/chemotherapy neoadjuvant							
Yes	1724	38.0	4819	40.7	6543	39.9	< 0.001
No	2816	62.0	7019	59.3	9835	60.1	
Total	4540	100.0	11838	100.0	16378	100.0

**Table 2:** Multivariable binary logistic regression on the likelihood of undergoing laparoscopic surgery for rectal cancer

	OR	95.0% CI for OR		*p* value
		Lower	Upper	
Sex				
Male	1.000			
Female	1.245	1.157	1.340	< 0.001
Age at diagnosis	0.982	0.978	0.985	< 0.001
Location rectum				
Lower rectum	1.000			
Middle rectum	1.202	1.073	1.346	< 0.002
Upper rectum	1.476	1.306	1.668	< 0.001
Unspecified	1.907	1.696	2.145	< 0.001
Stage UICC				
I	1.000			
II	0.742	0.677	0.813	< 0.001
III	0.845	0.776	0.920	< 0.001
Grading				
G1/2	1.000			
G3/4	0.826	0.739	0.924	< 0.001
Unspecified	1.494	1.301	1.714	< 0.001
Radio-/chemotherapy neoadjuvant				
Yes	1.000			
No	1.194	1.100	1.297	< 0.001

*OR* odds ratio, *CI* confidence interval

### Short-term survival

During a 90-day postoperative observation period, 1.7% of the laparoscopically treated patients vs. 3.1% of the open surgery patients died. Multivariable short-term mortality analysis delivered significantly better results for the laparoscopic approach (OR 0.658, 95% CI 0.526–0.822, *p *< 0.001; Table 3).

**Table 3:** Multivariable binary logistic regression concerning 90-day mortality for patients with rectal cancer (*N *= 16,977)

	OR	95.0% CI for OR		*p* value
		Lower	Upper	
Surgical approach				
Open	1.000			
Laparoscopic	0.658	0.526	0.822	< 0.001
Sex				
Male	1.000			
Female	0.659	0.551	0.788	< 0.001
Age at diagnosis	1.093	1.082	1.104	< 0.001
Location rectum				
Lower rectum	1.000			
Middle rectum	1.055	0.810	1.373	0.693
Upper rectum	0.816	0.614	1.085	0.161
Unspecified	0.907	0.688	1.196	0.490
Stage UICC				
I	1.000			
II	1.255	1.014	1.553	0.037
III	1.256	1.018	1.549	0.033
Grading				
G1/2	1.000			
G3/4	1.086	0.856	1.377	0.499
Unspecified	1.007	0.676	1.498	0.974
Radio-/chemotherapy neoadjuvant				
Yes	1.000			
No	1.644	1.327	2.037	< 0.001

*OR* odds ratio, *CI* confidence interval

### Long-term survival

**Fig. 3:**
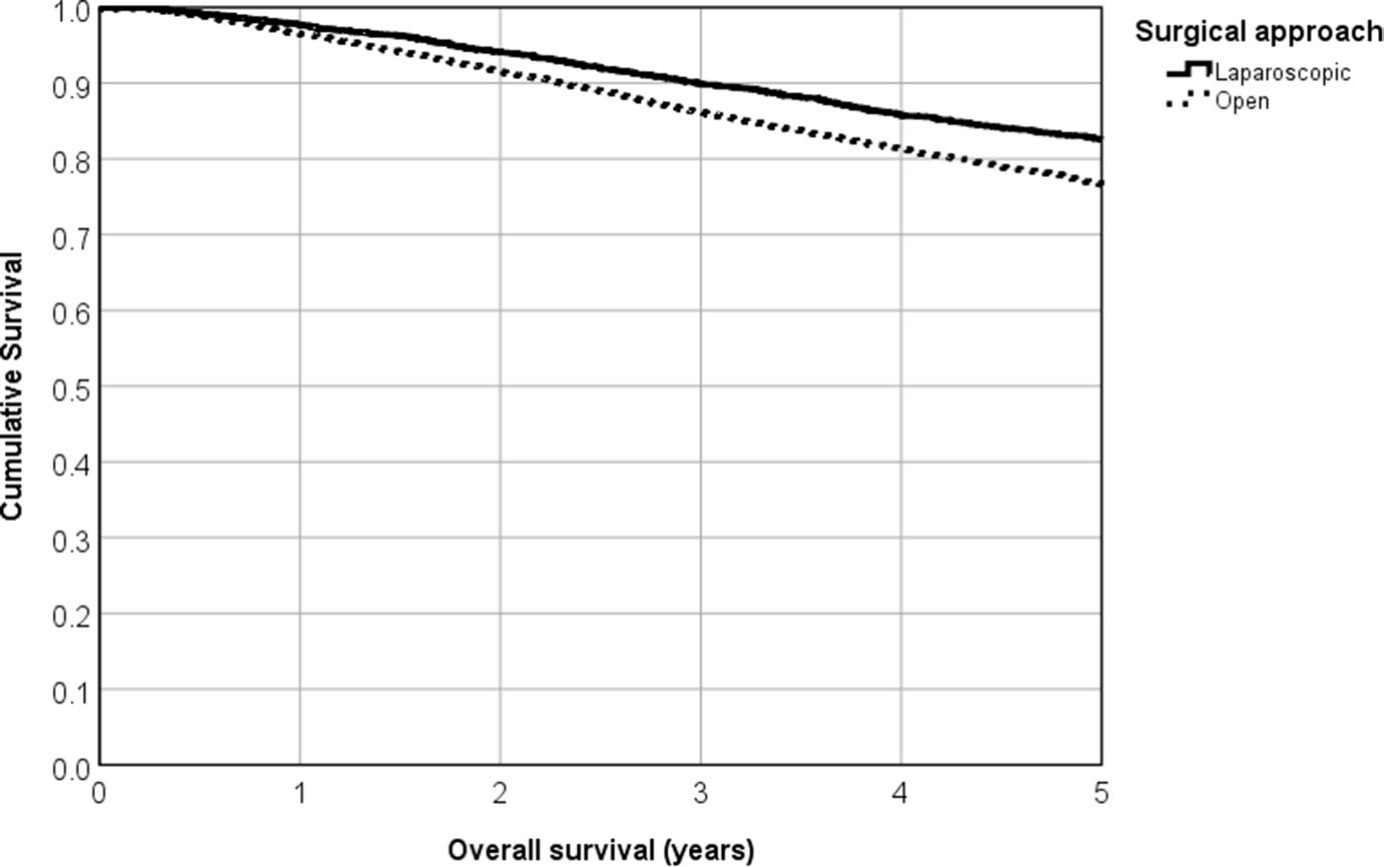
5-year cumulative overall survival rate for laparoscopic versus open surgery (82.6% vs. 76.6%, *p* < 0.001, Kaplan–Meier analysis)

**Fig. 4:**
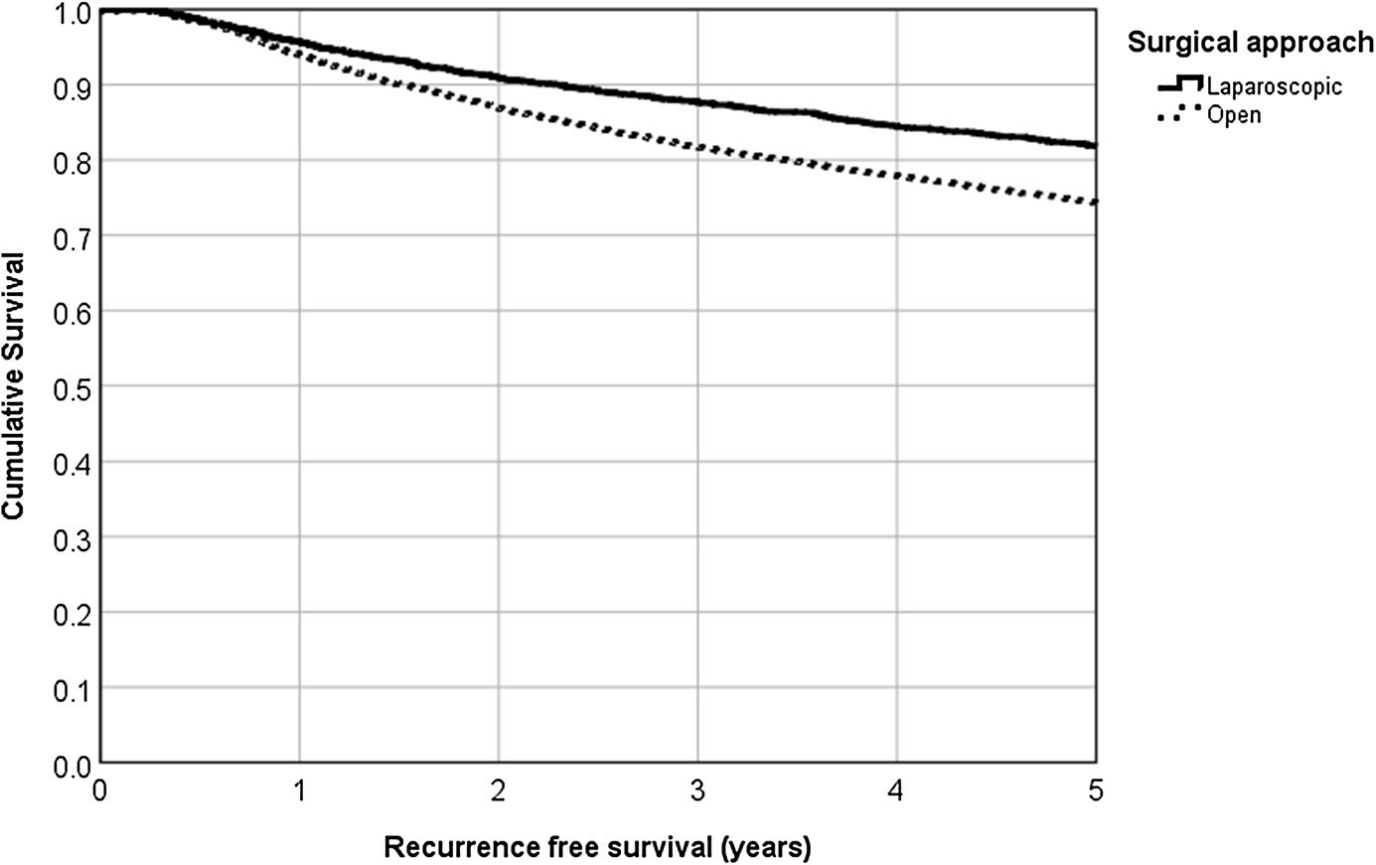
5-year cumulative recurrence-free survival rate for laparoscopic versus open surgery (81.8% vs. 74.3%, *p* < 0.001, Kaplan–Meier analysis)

Mean follow-up was 4.7 years (median 4.5 years). Both OS and RFS curves show better outcomes in favor of the laparoscopic approach (Figs. 3, 4). Comparing the 5-year survival rates, we found 82.6 vs. 76.6% for OS (logrank *p *< 0.001) and 81.8 vs. 74.3% for RFS (*p *< 0.001) for laparoscopic and open surgery, respectively. Relative survival rates also favor laparoscopy, with 95.7 vs. 93.3% (3-year survival), 93.1 vs. 88.4% (5-year survival), and significantly different survival curves (*p *= 0.012).

Univariable Cox regression disclosed significant superiority of the laparoscopic approach for rectal cancer, with HR 0.708 (95% CI 0.645–0.776, *p *< 0.001) for OS and HR 0.680 (95% CI 0.622–0.742, *p *< 0.001) for RFS. The effect was slightly diminished in multivariable regression after adjusting for sex, age of diagnosis, tumor location, UICC stage, grading, and radio-/chemotherapy, but still remained highly significant, with HR 0.819 (95% CI 0.747–0.899, *p *< 0.001) for OS and HR 0.770 (95% CI 0.705–0.842, *p *< 0.001) for RFS (Table 4).

**Table 4:** Multivariable Cox regressions: overall survival and recurrence-free survival of patients with rectal cancer

	HR	95.0% CI for HR		*p* value
		Lower	Upper	
*Overall survival*				
Surgical approach				
Open	1.000			
Laparoscopic	0.819	0.747	0.899	< 0.001
Sex				
Male	1.000			
Female	0.803	0.745	0.864	< 0.001
Age at diagnosis	1.063	1.059	1.067	< 0.001
Location rectum				
Lower rectum	1.000			
Middle rectum	0.993	0.895	1.101	0.890
Upper rectum	0.918	0.819	1.028	0.137
Unspecified	1.002	0.889	1.130	0.976
Stage UICC				
I	1.000			
II	1.681	1.524	1.855	< 0.001
III	2.276	2.077	2.495	< 0.001
Grading				
G1/2	1.000			
G3/4	1.192	1.085	1.310	< 0.001
Unspecified	0.976	0.828	1.150	0.771
Radio/chemotherapy neoadjuvant				
Yes	1.000			
No	1.163	1.071	1.264	< 0.001
*Recurrence-free survival*				
Surgical approach				
Open	1.000			
Laparoscopic	0.770	0.705	0.842	< 0.001
Sex				
Male	1.000			
Female	0.830	0.773	0.892	< 0.001
Age at diagnosis	1.052	1.048	1.055	< 0.001
Location rectum				
Lower rectum	1.000			
Middle rectum	0.945	0.856	1.044	0.265
Upper rectum	0.896	0.804	0.999	0.047
Unspecified	0.930	0.829	1.042	0.212
Stage UICC				
I	1.000			
II	1.647	1.499	1.811	< 0.001
III	2.241	2.052	2.447	< 0.001
Grading				
G1/2	1.000			
G3/4	1.195	1.091	1.309	< 0.001
Unspecified	0.976	0.836	1.141	0.763
Radio-/chemotherapy neoadjuvant				
Yes	1.000			
No	1.166	1.077	1.263	< 0.001

*HR* hazard ratio, *CI* confidence interval

Furthermore, our results for laparoscopic vs. open surgery remained stable after propensity score matching (1:2 nearest neighbor matching with caliper 0.2, balancing for sex, age, tumor location, stage, grading, and perioperative therapy), which rendered 4534 patients with laparoscopic and 8817 patients with open surgery. Specifically, the results for laparoscopic surgery in univariable analysis after propensity matching was HR 0.766 (95% CI 0.696–0.843, *p* < 0.001) for OS and HR 0.731 (95% CI 0.666–0.801, *p* < 0.001) for RFS. The multivariable analysis delivered HR 0.812 (95% CI 0.738–0.894, *p* < 0.001) for OS and HR 0.764 (95% CI 0.697–0.838, *p* < 0.001).

Upon performing multivariable Cox regression subgroup analyses for UICC stages I–III in the collective of 16,378 cases, a tendency toward better OS and RFS with the laparoscopic approach is seen in every stage, but results only remain significant for OS in stage III and for RFS in stages II and III (Table 5). Subgroup analysis on tumor location shows a statistically significant advantage of the laparoscopic approach for all rectum thirds in terms of OS (lower rectum HR 0.727, 95% CI 0.569–0.929, *p *= 0.011; middle rectum HR 0.837, 95% CI 0.717–0.977, *p *= 0.024; upper rectum HR 0.818, 95% CI 0.695–0.962, *p *= 0.015) and RFS (lower rectum HR 0.697, 95% CI 0.554–0.877, *p *= 0.002; middle rectum HR 0.787, 95% CI 0.677–0.915, *p *= 0.002; upper rectum HR 0.773, 95% CI 0.661–0.905, *p *= 0.001; Table 5). Multivariable Cox regression subgroup analysis for the different sexes also delivers favorable results for laparoscopy. Both men and women obtain better OS (men: HR 0.817, 95% CI 0.727–0.918, *p *= 0.001; women: HR 0.828, 95% CI 0.712–0.963, *p *= 0.015) and RFS (men: HR 0.778, 95% CI 0.696–0.871, *p *< 0.001; women: HR 0.760, 95% CI 0.657–0.879, *p *= 0.001; Table 5) with laparoscopic treatment.

**Table 5:** Hazard ratios for laparoscopic versus open surgery from multivariable Cox regression stratified for UICC stage, tumor locations, and sex

Survival	Stratification variable	HR	95.0% CI for HR		*p* value
			Lower	Upper	
Overall survival	UICC I	0.867	0.719	1.045	0.134
	UICC II	0.872	0.735	1.034	0.116
	UICC III	0.778	0.678	0.892	< 0.001
	Upper rectum	0.818	0.695	0.962	0.015
	Middle rectum	0.837	0.717	0.977	0.024
	Lower rectum	0.727	0.569	0.929	0.011
	Male sex	0.817	0.727	0.918	0.001
	Female sex	0.828	0.712	0.963	0.015
Recurrence-free survival	UICC I	0.875	0.734	1.044	0.138
	UICC II	0.815	0.691	0.962	0.015
	UICC III	0.708	0.620	0.808	< 0.001
	Upper rectum	0.773	0.661	0.905	0.001
	Middle rectum	0.787	0.677	0.915	0.002
	Lower rectum	0.697	0.554	0.877	0.002
	Male sex	0.778	0.696	0.871	< 0.001
	Female sex	0.760	0.657	0.879	< 0.001

Adjustment for sex, age, tumor location, UICC stage, grading, and perioperative treatment

*HR* hazard ratio, *CI* confidence interval

## Discussion

Laparoscopy has been used increasingly over the past years, but data regarding the oncologic resection quality compared to open surgery are still scarce. This study aimed to the supplement findings of the few existing studies in order to obtain a better understanding of the effects of laparoscopy compared to open surgery as a treatment for rectal cancer. Very positive short-term effects and immediate advantages of the laparoscopic approach are described in various publications, such as reduced blood loss [19–26], shorter hospitalization time [20–32], faster bowel movement recovery [20, 22, 24, 25, 33], fewer complications [14, 19–21, 28, 34], a better view in the low pelvic area [21, 26, 35, 36], and a lower short-term mortality rate [28, 34, 37]; however, negative elements such as increased operation time [19, 22–25, 30–32] and higher costs [25] have also been reported. The prolonged operation time for laparoscopy seems to be due to the degree of experience, as there was no significant difference in a Chinese study where all patients were treated by the same two well-trained surgeons [26]. Short-term outcome components like these have not been examined in the present study, except for perioperative mortality. The 90-day mortality analysis could confirm the favorable results for short-term survival with laparoscopic treatment, as there were significantly less deaths in the minimally invasive group (1.7 vs. 3.1%, OR 0.658, *p *< 0.001). This also confirms the decision to exclude all patients who died within 90 days after the operation, in order to correct for the distorting effect of short-term incidents on long-term survival outcomes.

The aspect of long-term morbidity such as bowel obstruction and incisional and parastomal hernias within 5 years was examined in the COLOR II study, with the conclusion that both open and laparoscopic surgery deliver similar results [38]. The CLASSIC study trial (2005) reported increased positive circumferential resection margins (CRM) for laparoscopic anterior resections in rectal cancer patients compared to the open approach (12.4 vs. 6.3%) [39]. Even though these results were not statistically significant, oncologic resection equality has been questioned and more studies have been conducted on this issue. CRM was not found to be different in the COLOR II study (2013), with 3% incomplete resections for both surgical approaches. Furthermore, 3-year survival rates tended to favor laparoscopy, with 86.7 vs. 83.6% (difference 3.1 percentage points; 95% CI 1.6–7.8) [15], with the limitation of not being significant. The difference between the two surgical approaches becomes clearer when expanding the observation period, as was done in the present study. While other studies did not find a significant advantage for either of the techniques in terms of OS and RFS [15, 21, 31, 37, 40], the present study identified significant differences in both aspects. The favorable outcomes for laparoscopy retained significance after adjustment in multivariable Cox regression for OS in UICC stage III as well as for RFS in UICC stages II and III. The reasons for these observations slightly differing from others reported in the literature could be improvements in surgical techniques and materials in the past years, but also greater expertise of the operating surgeons. In Germany—the source of the data in the current study—the number of specialized cancer centers has increased massively since 2007. A German retrospective cohort study displayed that treatment of colorectal cancer in specialized cancer centers shows significantly superior survival rates compared to hospitals that have not been certified as a center. International studies also demonstrate the survival benefit for patients treated in such centers [41, 42]. The beneficial effects we found for laparoscopic surgery might also be partly explained by the findings of a study on stress biomarkers in colorectal resections. Cortisol, cortisone, and glucose decrease more slowly in open surgery than with the minimally invasive approach. This slow decrease is considered to have a negative impact on the long-term outcome [43].

### Limitations

Comorbidities doubtless have an impact on the short- and long-term outcome of surgery by increasing the all-cause mortality. They can also be a reason for incomplete resections leading to tumor-associated deaths, which we addressed by excluding patients with residual tumor after surgery. Nevertheless, a shortcoming of this study is not having included comorbidities in the multivariate analysis. Since comorbidities are linked to age, adjusting for such does not entirely rule out the effect on patients’ survival induced by comorbidities, but may do so partly [44]. There were insufficient data to assess the patients’ physical status, which would have been possible with the ASA or ECOG classification system that evaluates the patients’ fitness before surgery. Furthermore, it is likely that we were not able to detect all emergency operations in the dataset: this variable was not collected by every contributing registry for the complete study period and might be thus underrepresented. Although we could not identify all emergency operations, 399 cases with this status were successfully excluded.

## Conclusion

One of the strengths of this study is its extraordinarily large sample size, with 16,378 cases from 30 clinical registries in Germany. Relying on this sound database, we can state that laparoscopic surgery is somewhat superior to open surgery for rectal cancer in terms of short-term mortality, relative survival, OS, and RFS. It delivers superior results for 90-day mortality as well as for OS in UICC stage III and RFS in stages II and III. Neither a significant negative trend that would argue against laparoscopy nor a negative tendency was found. Taking into account the results of other studies cited in this article, one can summarize that the laparoscopic approach is generally associated with favorable outcomes not only in terms of oncologic safety, OS, and recurrence-free survival, but it is also linked to positive short-term effects. This indicates that the laparoscopic approach performed by well-trained surgeons should be considered a first-choice treatment for rectal cancer.

## References

[1] Robert Koch-Institut Bericht zum Krebsgeschehen in Deutschland 2016

[2] Jemal A, Ward EM, Johnson CJ et al. (2017) Annual Report to the Nation on the Status of Cancer, 1975-2014, Featuring Survival. J Natl Cancer Inst 109(9). 10.1093/jnci/djx030 10.1093/jnci/djx030 PMC5409140 28376154

[3] Reynolds W (2001). The first laparoscopic cholecystectomy. JSLS.

[4] Cheong C, Kim NK (2017). Minimally Invasive Surgery for Rectal Cancer: current Status and Future Perspectives. Indian J Surg Oncol.

[5] Jacobs M, Verdeja JC, Goldstein HS (1991). Minimally invasive colon resection (laparoscopic colectomy). Surg Laparosc Endosc.

[6] Juo Y-Y, Hyder O, Haider AH (2014). Is minimally invasive colon resection better than traditional approaches?: first comprehensive national examination with propensity score matching. JAMA Surg.

[7] Mamidanna R, Burns EM, Bottle A (2012). Reduced risk of medical morbidity and mortality in patients selected for laparoscopic colorectal resection in England: a population-based study. Arch Surg.

[8] Panis Yves, Maggiori Léon, Caranhac Gilbert, Bretagnol Frederic, Vicaut Eric (2011). Mortality After Colorectal Cancer Surgery. Annals of Surgery.

[9] Benz S, Barlag H, Gerken M (2017). Laparoscopic surgery in patients with colon cancer: a population-based analysis. Surg Endosc.

[10] Völkel Vinzenz, Draeger Teresa, Gerken Michael, Klinkhammer-Schalke Monika, Fürst Alois (2018). Long-term oncologic outcomes after laparoscopic vs. open colon cancer resection: a high-quality population-based analysis in a Southern German district. Surgical Endoscopy.

[11] Ma Y, Yang Z, Qin H (2011). A meta-analysis of laparoscopy compared with open colorectal resection for colorectal cancer. Med Oncol.

[12] Ohtani H, Tamamori Y, Arimoto Y (2011). A Meta-Analysis of the Short- and Long-Term Results of Randomized Controlled Trials That Compared Laparoscopy-Assisted and Conventional Open Surgery for Colorectal Cancer. J Cancer.

[13] Wang C-L, Qu G, Xu H-W (2014). The short- and long-term outcomes of laparoscopic versus open surgery for colorectal cancer: a meta-analysis. Int J Colorectal Dis.

[14] Chen H, Ma B, Gao P (2017). Laparoscopic intersphincteric resection versus an open approach for low rectal cancer: a meta-analysis. World J Surg Oncol.

[15] Bonjer HJ, Deijen CL, Abis GA (2015). A randomized trial of laparoscopic versus open surgery for rectal cancer. N Engl J Med.

[16] Draeger Teresa, Völkel Vinzenz, Gerken Michael, Klinkhammer-Schalke Monika, Fürst Alois (2018). Long-term oncologic outcomes after laparoscopic versus open rectal cancer resection: a high-quality population-based analysis in a Southern German district. Surgical Endoscopy.

[17] Human Mortality Database: Database. University of California, Berkeley (USA), and Max Planck Institute for Demographic Research (Germany). Available at: https://www.mortality.org/. Accessed 08 Oct 2018

[18] Pohar M, Stare J (2006). Relative survival analysis in R. Comput Methods Programs Biomed.

[19] McKay GD, Morgan MJ, Wong S-KC (2012). Improved short-term outcomes of laparoscopic versus open resection for colon and rectal cancer in an area health service: a multicenter study. Dis Colon Rectum.

[20] Tong G, Zhang G, Liu J (2017). A meta-analysis of short-term outcome of laparoscopic surgery versus conventional open surgery on colorectal carcinoma. Medicine (Baltimore).

[21] Lujan J, Valero G, Biondo S (2013). Laparoscopic versus open surgery for rectal cancer: results of a prospective multicentre analysis of 4,970 patients. Surg Endosc.

[22] Bedirli A, Salman B, Yuksel O (2014). Laparoscopic versus Open Surgery for Colorectal Cancer: a Retrospective Analysis of 163 Patients in a Single Institution. Minim Invasive Surg.

[23] Matsuhashi N, Takahashi T, Tanahashi T (2017). Safety and feasibility of laparoscopic intersphincteric resection for a lower rectal tumor. Oncol Lett.

[24] van der Pas MH, Haglind E, Cuesta MA (2013). Laparoscopic versus open surgery for rectal cancer (COLOR II): short-term outcomes of a randomised, phase 3 trial. Lancet Oncol.

[25] Chen K, Zhang Z, Zuo Y (2014). Comparison of the clinical outcomes of laparoscopic-assisted versus open surgery for colorectal cancer. Oncol Lett.

[26] Yang Q, Xiu P, Qi X (2013). Surgical margins and short-term results of laparoscopic total mesorectal excision for low rectal cancer. JSLS.

[27] Ströhlein Michael A., Grützner Klaus-Uwe, Jauch Karl-Walter, Heiss Markus M. (2008). Comparison of Laparoscopic vs. Open Access Surgery in Patients with Rectal Cancer: A Prospective Analysis. Diseases of the Colon & Rectum.

[28] Kolfschoten NE, van Leersum NJ, Gooiker GA (2013). Successful and safe introduction of laparoscopic colorectal cancer surgery in Dutch hospitals. Ann Surg.

[29] Nussbaum Daniel P., Speicher Paul J., Ganapathi Asvin M., Englum Brian R., Keenan Jeffrey E., Mantyh Christopher R., Migaly John (2014). Laparoscopic Versus Open Low Anterior Resection for Rectal Cancer: Results from the National Cancer Data Base. Journal of Gastrointestinal Surgery.

[30] Park JS, Choi G-S, Jun SH (2011). Laparoscopic versus open intersphincteric resection and coloanal anastomosis for low rectal cancer: intermediate-term oncologic outcomes. Ann Surg.

[31] Chi P, Huang S-H, Lin H-M (2015). Laparoscopic transabdominal approach partial intersphincteric resection for low rectal cancer: surgical feasibility and intermediate-term outcome. Ann Surg Oncol.

[32] Yamamoto S, Fujita S, Akasu T (2011). Short-term outcomes of laparoscopic intersphincteric resection for lower rectal cancer and comparison with open approach. Dig Surg.

[33] Chen K, Cao G, Chen B (2017). Laparoscopic versus open surgery for rectal cancer: a meta-analysis of classic randomized controlled trials and high-quality Nonrandomized Studies in the last 5 years. Int J Surg.

[34] Mroczkowski P, Hac S, Smith B (2012). Laparoscopy in the surgical treatment of rectal cancer in Germany 2000-2009. Colorectal Dis.

[35] Hamada Madoka, Matsumura Tomonori, Matsumoto Tomoko, Teraishi Fuminori, Ozaki Kazuhide, Nakamura Toshio, Fukui Yasuo, Nishioka Yutaka, Taniki Toshikatu, Horimi Tadashi (2010). Advantages of the laparoscopic approach for intersphincteric resection. Surgical Endoscopy.

[36] Huh JW (2014). Minimally invasive techniques for an intersphincteric resection and lateral pelvic lymph node dissection in rectal cancer. Ann Coloproctol.

[37] Green BL, Marshall HC, Collinson F (2013). Long-term follow-up of the Medical Research Council CLASICC trial of conventional versus laparoscopically assisted resection in colorectal cancer. Br J Surg.

[38] Petersson Josefin, Koedam Thomas W., Bonjer H. Jaap, Andersson John, Angenete Eva, Bock David, Cuesta Miguel A., Deijen Charlotte L., Fürst Alois, Lacy Antonio M., Rosenberg Jacob, Haglind Eva (2019). Bowel Obstruction and Ventral Hernia After Laparoscopic Versus Open Surgery for Rectal Cancer in A Randomized Trial (COLOR II). Annals of Surgery.

[39] Guillou PJ, Quirke P, Thorpe H (2005). Short-term endpoints of conventional versus laparoscopic-assisted surgery in patients with colorectal cancer (MRC CLASICC trial): multicentre, randomised controlled trial. The Lancet.

[40] Jeong S-Y, Park JW, Nam BH (2014). Open versus laparoscopic surgery for mid-rectal or low-rectal cancer after neoadjuvant chemoradiotherapy (COREAN trial): survival outcomes of an open-label, non-inferiority, randomised controlled trial. Lancet Oncol.

[41] Onega T, Duell EJ, Shi X (2009). Influence of NCI cancer center attendance on mortality in lung, breast, colorectal, and prostate cancer patients. Med Care Res Rev.

[42] Beckmann MW, Brucker C, Hanf V (2011). Quality assured health care in certified breast centers and improvement of the prognosis of breast cancer patients. Onkologie.

[43] Netto Jeffrey, Jansen-Winkeln Boris, Thieme René, Eckardt Jan, Ju Bae Yoon, Willenberg Anja, Huppert Sabine, Lyros Orestes, Niebisch Stefan, Allecke Friederike, Kreuser Nicole, Kratzsch Jürgen, Kaiser Thorsten, Ceglarek Uta, Thiery Joachim, Gockel Ines (2018). Stress biomarkers in minimally invasive and conventional colorectal resections. Acta Chirurgica Belgica.

[44] Piccirillo JF, Vlahiotis A, Barrett LB (2008). The changing prevalence of comorbidity across the age spectrum. Crit Rev Oncol Hematol.

